# Liver resection combined with inferior vena cava resection and reconstruction using artificial vascular graft: A literature review

**DOI:** 10.1002/ags3.12068

**Published:** 2018-04-15

**Authors:** Yoshito Tomimaru, Hidetoshi Eguchi, Hiroshi Wada, Yuichiro Doki, Masaki Mori, Hiroaki Nagano

**Affiliations:** ^1^ Department of Gastroenterological Surgery Graduate School of Medicine Osaka University Suita Japan; ^2^ Department of Gastroenterological Surgery Toyonaka Municipal Hospital Toyonaka Japan; ^3^ Department of Gastroenterological Surgery Osaka International Cancer Institute Osaka Japan; ^4^ Department of Gastroenterological, Breast and Endocrine Surgery Yamaguchi University Graduate School of Medicine Ube Japan

**Keywords:** artificial vascular graft, inferior vena cava, liver resection, reconstruction

## Abstract

In cases where liver tumors invade the inferior vena cava (IVC), IVC resection along with liver resection may be needed to effect a cure. Furthermore, if the IVC defect is large, IVC reconstruction with vascular graft after resection is required. There are limited reports of cases of IVC reconstruction using a graft. By reviewing data from the literature of previous studies, the present study was aimed at investigating the surgical outcomes of liver resection with IVC resection and reconstruction using an artificial vascular graft. PubMed was searched for previous articles reporting cases with the combined surgery. The search was limited to articles in English, and cases with exceptional surgeries such as in situ cold perfusion, and ante situm and ex vivo techniques were excluded from this study. Surgical outcomes of the extracted cases were investigated. Cases dealt only with primary closure after IVC resection, and those in which the IVC tumor thrombus was treated by opening the IVC wall, removing the thrombus and then closing the IVC without wall excision were not included in this study. The literature search identified 13 studies, including 111 cases. Operative mortality in the reported cases was 8.1% (9 out of 111 cases). Thrombus in the artificial vascular graft was observed in two cases, and patency of the graft during the follow‐up period was confirmed in 109 of the 111 cases (98.2%). These results suggested that the surgical outcomes of liver resection combined with IVC resection and reconstruction using the artificial vascular graft were favorable.

## 1 INTRODUCTION

Liver resection has frequently been the only curative treatment for liver tumors such as hepatocellular carcinoma (HCC), intrahepatic cholangiocarcinoma (ICC), and liver metastasis from various types of cancers (LM). These tumors sometimes directly invade the inferior vena cava (IVC). In cases with IVC invasion, liver resection combined with IVC reconstruction provides the only chance for a cure. Furthermore, if the area affected by the IVC resection is large, a vascular graft is required, although the graft is not necessarily needed in cases with a smaller defect area.^1^, ^2^ The surgery—liver resection combined with IVC resection and reconstruction—has been considered challenging and associated with a high incidence of surgery‐related complications and mortality. However, recent progress in surgical techniques and perioperative management may lead to improvement of clinical outcomes of the surgery.^3^ This background suggests the necessity for investigation of the surgery, especially focusing on surgical procedure and outcome, but there are limited reports of cases with liver resection and IVC reconstruction. Thus, in the present study, with an aim to examine surgical outcomes of the combined surgery, we reviewed previous studies through a literature search, regarding cases with liver resection combined with IVC resection and reconstruction. Based on the finding that as a vascular graft, artificial vascular graft seems popular, herein we focused on cases where an artificial vascular graft was used for IVC reconstruction after liver resection and IVC resection.

## 2 METHODS

**Figure 1:**
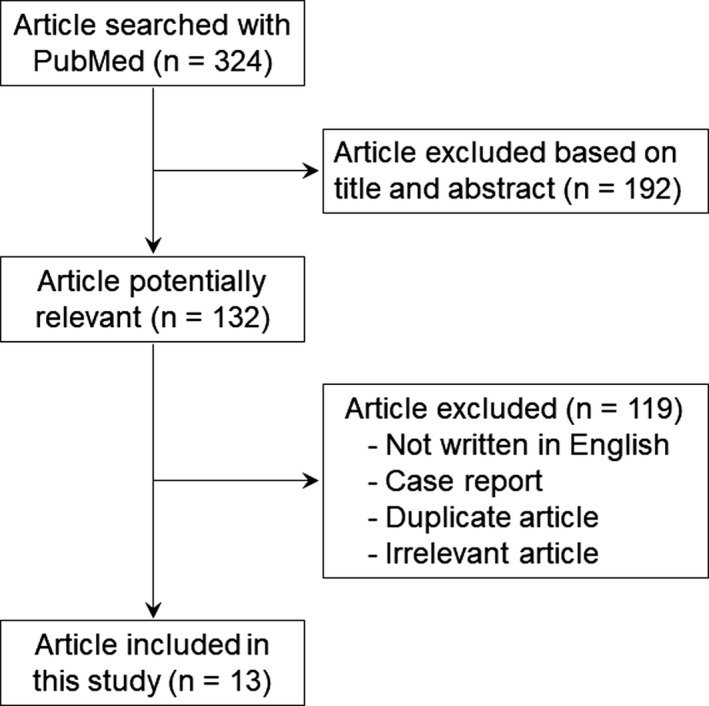
Flowchart representing the selection of articles for the present study. The search was carried out using specific terms with PubMed. The 324 searched articles were first screened by title and abstract of the articles. Then, the remaining 132 potentially relevant articles were further assessed for their relevance. Finally, 13 articles were selected for this study

In December 2017, PubMed was searched for previous articles reporting cases with liver resection combined with IVC resection and reconstruction using artificial vascular graft. The search was carried out using the terms “liver resection” (or “hepatic resection”, or “hepatectomy”) in combination with “inferior vena cava” (or “inferior caval vein”, or “IVC”) and “reconstruction” (or “graft”, or “ePTFE”, or “replacement”). The searched articles were first screened by title of the articles. Then, the search was limited to articles in English. “Case report” articles and duplicate articles were excluded from this study. Cases dealt only with primary closure after IVC resection, and those in which the IVC tumor thrombus was treated by opening the IVC wall, removing the thrombus and then closing the IVC without wall excision were not included in this study. Cases that received associated procedures such as in situ cold perfusion, and ante situm and ex vivo techniques were also excluded from this study. The remaining potentially relevant articles were further assessed for their relevance based on content of the articles. Thus, cases with liver resection combined with IVC resection and reconstruction using an artificial vascular graft were extracted from the final selected articles. Flowchart representing the selection of articles for this study is shown in Figure 1. In the cases, surgical outcomes especially in the incidence of operative mortality and postoperative complications, and patency of the vascular graft, were investigated.

## 3 RESULTS

Through the literature search, no systemic reviews were found and there were 13 study articles that met the inclusion criteria (Figure 1).^1^, ^2^, ^4^, ^5^, ^6^, ^7^, ^8^, ^9^, ^10^, ^11^, ^12^, ^13^, ^14^, ^15^, ^16^ The 13 studies are summarized in Table 1 and included 111 cases. Median number of cases in each study was four (range = 3‐33). The liver tumor to be resected at surgery in the 111 cases was as follows: HCC in 12 cases, ICC in 26 cases, LM in 19 cases, and others in 18 cases. Others included other liver tumors such as sarcoma and direct invasion to the liver from other organs. In a particular study,^15^ the liver tumor in 33 cases was diagnosed as HCC, ICC, LM or others, but distribution of the disease was unknown. In another report, the disease of three cases was not described.^8^

**Table 1:** Reports of cases from previous studies of liver resection combined with IVC resection and reconstruction using artificial vascular graft

No.	Authors	Year	No. cases	Diagnosis	Operative mortality (%)	Cause of mortality	Patency (%)
1	Huguet et al^5^	1995	3	LM = 1, Others = 2	1 (33.3)	Liver failure	3 (100)
2	Ohwada et al^6^	1999	4	HCC = 3, LM = 1	1 (25.0)	Liver failure	4 (100)
3	Madariaga et al^2^	2000	7	ICC = 3, Others = 4	1 (14.2)	Liver failure	7 (100)
4	Hardwigsen et al^7^	2001	3	ICC = 2, Others = 1	0 (0)	N/A	3 (100)
5	Maeba et al^8^	2001	3	N/A	0 (0)	N/A	3 (100)
6	Arii et al^9^	2003	11	HCC = 3, ICC = 4, LM = 2, Others = 2	1 (9.1)	GVHD	11 (100)
7	Sarmiento et al^1^, ^10^	2003	18	HCC = 2, ICC = 9, LM = 5, Others = 2	1 (5.5)	Bleeding	16 (88.9)
8	Azoulay et al^12^	2006	4	ICC = 3, Others = 1	0 (0)	N/A	4 (100)
9	Ohwada et al^14^	2007	3	Others = 3	0 (0)	N/A	3 (100)
10	Delis et al^13^	2007	12	HCC = 4, ICC = 2, LM = 6	0 (0)	N/A	12 (100)
11	Malde et al^4^	2011	4	LM = 2, Others = 2	1 (25.0)	Multiple organ failure	4 (100)
12	Hemming et al^11^, ^15^	2013	33	HCC, ICC, LM, Others^a^	3 (9.1)	N/A	33 (100)
13	Orimo et al^16^	2014	6	ICC = 3, LM = 2, Others = 1	0 (0)	N/A	6 (100)
	Total		111	HCC = 12, ICC = 26, LM = 19, Others = 18	9 (8.1)		109 (98.2)

Numbers of each disease are unknown.

GVHD, graft versus host disease; HCC, hepatocellular carcinoma; ICC, intrahepatic cholangiocarcinoma; IVC, inferior vena cava; LM, liver metastasis, N/A, not applicable.

**Figure 2:**
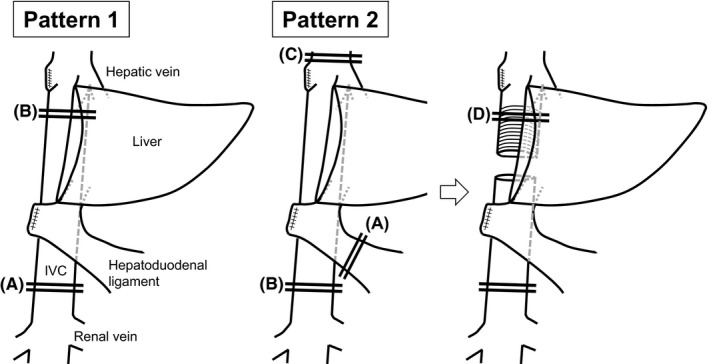
Patterns of clamping for inferior vena cava (IVC) resection and reconstruction. There were two patterns of clamping for IVC resection and reconstruction based on IVC involvement, as shown in these representative intraoperative images following right hemihepatectomy. Pattern 1 was adopted in cases where IVC involvement was located below the root of the hepatic veins, allowing for placement of the IVC clamp below the hepatic veins. In this pattern, clamp placement was below the IVC wall involved with the tumor (A), and on the IVC below the root of the hepatic veins (B). Pattern 2 was adopted in cases where the IVC clamp below the hepatic veins was not possible as a result of tumor involvement. The total hepatic vascular exclusion (THVE) technique was used for the clamp in these cases, with clamping of the hepatoduodenal ligament (A), the IVC below the IVC wall involved with the tumor (B), and the IVC above the root of the hepatic veins (C). The clamp above the root of the hepatic veins was repositioned below the root of the hepatic veins (D) for unclamping of the hepatoduodenal ligament for restoration of perfusion to the liver after the anastomosis of the cranial IVC side

In the searched studies, the surgical procedure was selected based on the extent of the tumor and residual liver function. Type of resection and repair was determined based on IVC involvement; when the involvement was small, IVC was reconstructed by primary repair or with a patch. For the patch, an artificial vascular graft, the expanded polytetrafluoroethylene (ePTFE) graft (Gore‐Tex; WL Gore & Associates, Inc., Flagstaff, AZ, USA), or bovine or horse pericardium were used. This review did not include cases with pericardium for patch repair. For a larger degree of IVC involvement, the entire circumference of the IVC was replaced with an artificial vascular graft. As the artificial vascular graft for the circumferential replacement of IVC, although Dacron graft (Hemashield; Meadox Medicals, Inc., Oakland, NJ, USA) was used in limited cases, ePTFE graft was used in most cases. There were two patterns of clamping reported based on the extent and location of IVC tumor involvement (Figure 2). The first pattern of clamping (pattern 1) was used in cases where IVC involvement with the tumor was located below the root of the hepatic veins and placement of the IVC clamp below the hepatic veins was allowed. In these cases, the IVC was clamped at the level of and also below the IVC wall involved with the tumor, allowing continued perfusion of the remnant liver during the IVC clamps. IVC resection and grafting was carried out while these clamps were in place. The second pattern (pattern 2) reported was adopted in cases where tumor involvement of the IVC did not allow for placement of the IVC clamp below the hepatic veins as a result of involvement with the tumor. In these cases, in addition to the clamp below the IVC wall involved with the tumor, the IVC above the root of the hepatic veins and the hepatoduodenal ligament were clamped, which is known as total hepatic vascular exclusion (THVE).^17^, ^18^ During THVE, the IVC was resected and reconstructed. After the reconstruction, the clamp above the root of the hepatic veins was repositioned below the root of the hepatic veins, allowing unclamping of the hepatoduodenal ligament for restoration of perfusion to the liver and minimization of liver ischemic time. Then, as with the first pattern, the caudal IVC side was anastomosed to the graft. For the reconstruction procedure in both patterns, anastomosis of the cranial IVC side was first carried out. Then, air was removed from the vascular graft by using blood backflow through the graft, followed by completion of the anastomosis of the caudal IVC side with the graft.

Surgical outcomes in each extracted study are also shown in Table 1. Postoperative complications were reported, but the definition of postoperative complication was not described. Operative mortality was found in nine cases (8.1%) out of 111 cases reported in the studies. Cause of the operative mortality was clearly described in six cases out of the nine cases; liver failure in three cases, multiple organ failure in one case, graft versus host disease in one case, and bleeding in one case. Thrombus in the vascular graft was observed in two cases and, therefore, patency of the artificial graft during follow‐up period was confirmed in 109 cases (98.2%) of the 111 cases.

## 4 DISCUSSION

The literature review showed that, in previous studies, operative mortality rate of liver resection combined with IVC resection and reconstruction using artificial vascular graft was 8.1%. Incidence of postoperative complications was undetermined because of unclear definitions of complications. In the previous studies, patency of the artificial vascular graft was 98.2%. We also experienced 12 cases of combined surgery, and summary of these cases showed that rates of mortality and postoperative complications defined as ≥grade III of the Clavien‐Dindo classification were 0% and 16.7%, respectively, and that the patency of the artificial vascular graft was 100% (unpublished data),^19^ which suggests not only consistency with the review results, but also feasibility of the surgery. To the best of our knowledge, there have been no studies strictly comparing surgical outcomes between cases of liver resection with and without IVC resection and reconstruction using the artificial vascular graft. However, it is well known that, even with recent progress in surgical techniques and perioperative management, liver resection combined with IVC resection and reconstruction using the artificial vascular graft is potentially associated with increased risk for morbidity and mortality compared to liver resection without vascular grafting. For example, according to National Clinical Database (NCD) in Japan (http://www.ncd.or.jp), which is a nationwide prospective registry linked to the surgical board certification system,^20^ patients undergoing hepatectomy with vascular reconstruction were reported to have a higher mortality than those without vascular reconstruction (odds ratio, 3.84 at 30‐days mortality, 2.96 at operative mortality), although these data include not only IVC reconstruction but also reconstruction of other vessels. Furthermore, although the 13 previous studies did show that the incidences of morbidity and mortality were not high, there may have been “publication bias”, leading to overestimation of the positivity of the outcomes. Taking these findings into consideration, feasibility of the combined surgery would be expected to be validated through comparative studies with more cases in the future. However, regardless of the type of cancer, patients with cancers involving the IVC have an extremely poor prognosis. For example, untreated ICC cases have only a median survival of approximately 3 months and chemotherapy does not offer a curative option.^21^ However, this case series contained relatively long‐term survival and previous studies contained cases with greater than 5‐year survival rates.^15^, ^16^ This prognostic information suggests a survival benefit of liver resection combined with IVC resection and subsequent reconstruction using the artificial vascular graft. When determining indication for the combined surgery, surgeons should consider both aspects of the combined surgery: the aforementioned potential operative risk and survival benefit.

Several concerns have been discussed regarding the procedure of liver resection combined with IVC resection and reconstruction using an artificial vascular graft. First, there is a problem regarding the necessity for IVC reconstruction after its resection. Although it is well known that IVC blood flow is responsible for renal insufficiency and lower extremity edema, IVC reconstruction does not seem feasible in cases with infrarenal IVC invasion with adequate collateralization of blood flow. However, this is not the case with suprarenal IVC invasion, which is the most common in cases with IVC invasion by liver tumors. Determining which patients will tolerate IVC resection without the previously mentioned problems is difficult. Reconstruction would be considered when suprarenal invasion is noted. The second problem is the necessity for antithrombotic therapy after the reconstruction. In more than half of the previous studies, antithrombotic therapy was given after the reconstruction, but no clear criteria for giving antithrombotic therapy were commented upon in each study. Antithrombotic therapy was carried out mainly by using heparin, although there were several cases where nafamostat mesilate was used with heparin, and those where aspirin was used after the heparin dose. This prevalence of antithrombotic therapy might be as a result of a study showing that, in two cases where thrombus was identified in the graft, antithrombotic therapy had been discontinued. However, necessity for antithrombotic therapy after the reconstruction has not been examined in previous studies with high level evidence such as prospective studies comparing outcomes between cases with and without antithrombotic therapy. Furthermore, this concern should be discussed considering also the abovementioned necessity for IVC reconstruction after the resection. Thus, this problem seems complicated, although it will ideally be solved in the future. Last, there have been other proposed procedures such as in situ cold perfusion,^4^, ^15^, ^22^, ^23^ and ante situm,^4^, ^24^, ^25^ and ex vivo techniques.^4^, ^15^, ^22^, ^23^ For example, the ex vivo technique interrupts and transects major inflow and outflow of the liver, as well as the bile duct, and removes the liver from the abdomen. On a back table, the liver remains under cold perfusion while tumor resection is carried out, followed by reimplantation.^15^ These procedures are effective for patients where obtaining tumor‐free margins, in situ, is not possible, but they are technically demanding complicated procedures and associated with high surgical stress and operative risks. Therefore, their indication should be strictly determined.

In summary, surgical outcomes of liver resection combined with IVC resection and reconstruction using an artificial vascular graft were investigated based on a literature review of previous studies. The results of the investigation showed that the outcomes were favorable, suggesting feasibility of the combined surgery.

## DISCLOSURE

Conflicts of interest: Authors declare no conflicts of interest for this article.
